# Integrated Transcriptomic and Network Analysis Identifies Diagnostic and Immune-Associated Hub Genes in Urothelial Carcinoma

**DOI:** 10.3390/biomedicines14102391

**Published:** 2026-10-19

**Authors:** Shaza N. Alkhatib, Ammena Y. Binsaleh, Nawal Al-Hoshani, Tahani Bakhsh, Huda A. Alqahtani, Tariq Aziz

**Affiliations:** 1 Department of Biological Sciences, College of Sciences and Arts Khulais, University of Jeddah, Jeddah 21959, Saudi Arabia; 2 Department of Pharmacy Practice, College of Pharmacy, Princess Nourah bint Abdulrahman University, P.O. Box 84428, Riyadh 11671, Saudi Arabia; 3 Department of Biology, College of Science, Princess Nourah bint Abdulrahman University, P.O. Box 84428, Riyadh 11671, Saudi Arabia; 4 Department of Biological Science, College of Science, University of Jeddah, P.O. Box 80327, Jeddah 21589, Saudi Arabia; 5 Department of Zoology, College of Science, King Saud University, Riyadh 11495, Saudi Arabia; 6 Department of Biology, College of Science, University of Tabuk, Tabuk 71491, Saudi Arabia; 7 Molecular Microbiology and Infectious Diseases Research Unit, University of Tabuk, Tabuk 71491, Saudi Arabia

**Keywords:** urothelial carcinoma, IL-6/JAK/STAT signaling, hub genes, diagnostic biomarkers, immune infiltration

## Abstract

**Background:** Urothelial carcinoma exhibits molecular heterogeneity, high recurrence, and progression rates, and is poorly diagnosed and treated. Transcriptomic and systems-biology analyses can detect dysregulated genes, signaling pathways, and interactions of immune cells. Proposed biomarkers, however, have not been validated, evaluated, and confirmed to be cell-type specific, limiting their potential for clinical translation. **Methods:** The transcriptomic data in GSE13507 were used to identify features of differential expression, functional enrichment, protein interaction network, and hub genes. Findings were assessed by using external validation, ROC and machine learning models, immune-infiltration analysis, single-cell expression profiling, and survival analysis. **Results:** In total, 7459 differentially expressed probes were detected, and pathways involved in immunity and cytokines were noted to be enriched. The best diagnostic performance was observed with IL6ST (AUC 0.927), LIFR (AUC 0.921), and JAK1 (AUC 0.843). We found a correlation between hub genes and infiltration of immune cells, and a difference in single-cell distribution; however, none of them was shown to have a significant association with overall survival. **Conclusions:** The IL-6/JAK/STAT hub gene network was highly diagnostic and immune-relevant in urothelial carcinoma and had limited prognostic significance. The three most promising candidates were IL6ST, LIFR, and JAK1. The genes identified in this study could be validated in tumor-normal pairs and in prospective clinical samples of future studies, accompanied by protein levels and functional studies.

## 1. Introduction

Urothelial carcinoma (UC) is reported to be one of the most common forms of bladder cancer. Due to its progression to an invasive form, it is a clinical burden on the healthcare system and is considered to be a medical challenge [1]. Because of its tendency to recurrence and progression, particularly the transition from non-muscle-invasive to muscle-invasive disease, there are serious difficulties in treatment. This transition is also associated with poor prognosis in patients, affecting both survival and quality of life. Thus, it is essential to achieve a better understanding of the underlying biology in order to improve clinical management and to provide more effective diagnostic and therapeutic tools [2,3]. The molecular mechanism of UC is complex and involves multiple dysregulated genes and signaling pathways. The development of tumors is assisted by interactions in the tumor microenvironment and genetic alterations [4]. In disease progression, the immune-related pathways and cytokine-mediated pathways play an important and central role. Due to the availability of high-throughput transcriptomic data, the identification of differentially expressed genes in UC has become easier. Network-based analyses of protein–protein interactions can help identify hub genes potentially involved in tumor-associated biological processes [5]. For the computational exploration of UC biology, publicly available datasets like NCBI GEP and TCGA constitute a rich resource. The potential therapeutic interventions and precision medicine approaches can be accessible through the identification of key regulatory networks [6,7].

Urothelial carcinoma is the most frequent type of bladder cancer and is a significant clinical problem due to its high recurrence rate, frequent progression, and significant molecular variability. Cystoscopy, histopathology, and urine-based tests are commonly employed for diagnosis and surveillance, but can be invasive and expensive, and lack early detection sensitivity and the ability to accurately stratify patients. Therefore, elucidation of molecular mechanisms in the pathogenesis of UC is critical to the development of reliable biomarkers and new therapeutic targets [8,9,10]. Bladder cancer development has previously been identified from transcriptomic and systems-biology studies to have extensive alterations in gene expression, immune regulation, cytokine signaling, epithelial organization, and tumor–microenvironment interactions [11]. Differential expression analysis of genes and clinically relevant molecular signatures has been performed using public resources like GEO and TCGA. The IL-6/JAK/STAT pathway has been associated with inflammation, immune evasion, tumor growth, and resistance to therapy in many cancers, in particular. Network-based strategies with STRING and Cytoscape have also been widely used to find hub genes that may play central regulatory functions [12,13].

The recent studies on urothelial carcinoma have started employing bulk transcriptomics along with machine learning, immune infiltration, and single-cell or spatial transcriptomics to detect potential molecular biomarkers for diagnostics and prognosis. The inclusion of independent cohorts and experimentation to validate findings in some cases has further demonstrated the advancement in the field of computational biomarker discovery for bladder cancer. Nonetheless, there are still many issues in the process, such as signature dependence on cohorts, lack of generalization, questions about the cellular basis of bulk expression, and the frequent leap from computational association to biological/clinical relevance without validation. Hence, what is needed is not yet another isolated predictive model but an integrative analysis of the consistency of candidate biomarkers across biological/immunological/cellular/clinical perspectives [14,15,16].

Recent advances in computational oncology have made it possible to integrate bulk transcriptomic data with machine learning models, immune deconvolution algorithms, and single-cell resources to obtain a more comprehensive view of tumor biology. These integrated approaches may be able to determine genes that are simply differentially expressed, as well as genes with immune associations and cell-type-specific activity that are also likely to have diagnostic value. This is especially relevant in UC, where there is molecular heterogeneity and tumor microenvironment variation which can have a significant impact on disease behavior and therapy response [17,18]. Another crucial factor is the difference between diagnostic and prognostic biomarkers. Although a gene distinguishing tumor from normal tissue may be useful, it does not necessarily have an association with the survival of the patient because diagnosis and survival outcome represent different processes. Functional evaluation alone will not lead to over-interpretation and will allow the identification of more appropriate and useful biomarkers for the detection of tumors, patient stratification, or investigation for therapeutic purposes [19].

Most of the previous studies were conducted with patients from a single discovery cohort, were not externally validated, or did not validate the hub genes for diagnostic performance [20]. In the case of urothelial carcinoma, the interaction of IL-6/JAK/STAT-related genes and immune cell infiltration is also not completely elucidated. Furthermore, bulk transcriptomic data are unable to distinguish whether candidate genes come from malignant epithelial cells, immune cells, or stromal cells. These restrictions decrease confidence in their biological meaning and clinical use [21,22].

The current study identified and validated the important regulatory genes involved in the development of UC through an integrated bioinformatics approach. Combined with external-cohort validation, ROC analysis, multigene diagnostic modeling, immune-infiltration assessment, single-cell expression analysis, and survival evaluation, differential-expression analysis, functional enrichment, PPI-network construction, and hub gene selection were performed. It was thought that an IL-6/JAK/STAT-based gene network would exhibit consistent expression, diagnostic relevance, and biological relevance to the tumor immune microenvironment.

The study utilized GSE13507 as the discovery dataset for identification of dysregulated genes and enriched pathways. Then, among the candidate hub genes, these were assessed in an independent urothelial carcinoma cohort and evaluated by diagnostic and machine learning methods. Relationships with immune cells were evaluated by several deconvolution approaches, and single-cell resources were used to discover where they were found. Lastly, to differentiate between diagnostic relevance and prognostic significance, a survival analysis was undertaken.

The contribution of this study lies primarily in the integration of established computational approaches rather than the development of a new algorithm. In particular, the study prioritizes IL-6/JAK/STAT-related candidate biomarkers by means of differential expression, enrichment analysis, and network topology, and tests the reproducibility of expression of these biomarkers in another dataset of urothelial carcinoma patients. It also assesses diagnosis prediction capacity using ROC and machine learning-based models; and considers immune cell infiltration, single-cell, and survival analyses in order to analyze the context of these biomarkers.

## 2. Materials and Methods

### 2.1. Study Design and Data Sources

This integrated bioinformatics study used GSE13507 as the discovery cohort and GSE32894 as an independent cohort to assess candidate hub-gene expression and intertumoral variability. The GSE13507 was selected as the discovery set due to the presence of urothelial carcinoma and non-tumorous bladder specimens with appropriate expression values and clinical data relevant to differential expression analysis. On the other hand, GSE32894 was used as an independent set of urothelial carcinomas to test the reproducibility and variability between patients for the candidate genes. Preprocessing and normalization specific to each dataset were performed before further analyses, and the probes were mapped to gene names. Each set of data was analyzed separately and was not merged into one expression matrix; hence, no batch effect correction across the datasets was performed.

### 2.2. Discovery Dataset Pre-Processing and Differential Expression Analysis

The expression data were obtained from the National Center for Biotechnology Information (NCBI) Gene Expression Omnibus (GEO) (https://www.ncbi.nlm.nih.gov/geo, Accessed on 9 July 2026). Gene expression analysis was carried out using iDEP (https://bioinformatics.sdstate.edu/idep, Accessed on 9 July 2026) from GSE13507, where expression analysis was filtered, transformed, clustered hierarchically, and visualized using UMAP. Differential expression analysis was carried out using GEO2R using the expression dataset of GSE13507. A significant *p* value was set as <0.05 with |log2 fold change| ≥ 1. Multiple-testing corrections were carried out using the GEO2R tool (https://www.ncbi.nlm.nih.gov/geo/geo2r, accessed on 9 July 2026). Mapping from probes to gene names was done based on platform annotation. Since differential expression results were initially derived from probes, 7459 differentially expressed entities are referred to as differentially expressed probes and not genes unless gene-level consolidation is carried out [23].

### 2.3. Functional Enrichment, PPI Network and Hub Gene Identification

To functionally annotate the DEGs, the genes were analyzed using the Enrichr tool (https://maayanlab.cloud/enrichr, Accessed on 11 July 2026), which allowed the exploration of the GO (http://geneontology.org, Accessed on 11 July 2026) and KEGG 118.2 databases (https://www.genome.jp/kegg/pathway.html, Accessed on 11 July 2026) [24,25,26]. The genes were categorized based on their association with biological processes, molecular functions, and cellular components, which could include the adaptive immune response and cytokine receptor binding. To relate the DEGs to pathology and biological significance, the genes were cross-checked in various databases, including Jensen DISEASES (https://diseases.jensenlab.org, Accessed on 12 July 2026), which relates genes to pathology; the Human Phenotype Ontology (https://hpo.jax.org, Accessed on 12 July 2026), which relates genes to phenotypic characteristics; and TISSUES (https://tissues.jensenlab.org, Accessed on 13 July 2026), which relates genes to organ-specific expression [27,28].

The STRING database (https://string-db.org, Accessed on 12 July 2026) was utilized for humans to explore the physical and functional interactions of the DEGs. To construct the interaction network, we applied a minimum interaction score of 0.4, which maintains a medium confidence level in all protein interactions. The reliability of the interaction network was assessed by a PPI enrichment *p*-value to ensure that the proteins are more interconnected than expected by random chance [29].

The PPI network was imported into the Cytoscape 3.10.4 program (https://cytoscape.org, Accessed on 12 July 2026) to further investigate the topological features and clustering of the network [30]. To determine the most important nodes in the network, we used the CytoHubba 0.1 plugin (https://apps.cytoscape.org/apps/cytohubba, Accessed on 12 July 2026) and selected the proteins based on the Maximal Clique Centrality score, a tool that has been shown to effectively target the most important genes in a network [31]. Concurrently, the MCODE 2.0.3 plugin (https://apps.cytoscape.org/apps/mcode, Accessed on 13 July 2026) was used to detect functionally important clusters, or modules, that represent particular biological sub-processes regulated by the key regulators that we have identified. The PPI network was built using the STRING 12.0 server with a minimum interaction confidence score of 0.4 and then plotted using Cytoscape. Hub genes of interest were selected using the CytoHubba plugin on Cytoscape using Maximal Clique Centrality (MCC) ranking, whereas dense clusters of interconnected nodes in the network were determined using the MCODE algorithm. Genes with significant network centrality and/or involvement in densely interconnected modules in the network were selected for further analysis [32].

### 2.4. Independent External Validation of Hub Genes

The gene expression data and clinical data from the independent GEO cohort GSE32894 were downloaded and preprocessed to validate the diagnostic and prognostic values of the identified hub genes (IL6, IL6ST, JAK1, LIFR, OSM, CNTF, IL11, and IL27RA). For the establishment of the validation matrix, log_2_ transformation and probe-to-gene symbol annotation were performed. The efficacy of the diagnosis was assessed by Area Under the Curve (AUC) and Receiver Operating Characteristic (ROC) analysis. Moreover, multi-algorithm immune infiltration deconvolution, single-cell cellular distribution profiling, and Cox proportional hazards survival regression models were built to evaluate the immunological relevance and prognostic significance of the hub gene signature [33,34].

### 2.5. Diagnostic Model Development and Validation

To validate the diagnostic accuracy and clinical application of the identified IL-6/JAK/STAT signaling hub genes, a machine learning-based diagnostic prediction model was built based on the dataset cohort (GSE13507). Data from the expression was log transformed and mapped to target gene symbols (IL6, IL6ST, JAK1, LIFR, OSM, CNTF, IL11, IL27RA), and samples were stratified into tumor and normal diagnostic classes. A receiver operating characteristic (ROC) curve analysis of each biomarker was used to quantify the classification performance, and the Area Under the Curve (AUC) and corresponding 95% confidence intervals were calculated to assess diagnostic sensitivity and specificity of the biomarkers to identify the patients with UC from non-tumor controls. The machine learning analysis was performed as an exploratory assessment of the discriminatory potential of the selected hub genes rather than as a fully externally validated predictive model. Logistic regression and Random Forest analyses were used to examine the relative predictive contribution of the candidate genes, while ROC curves and AUC values were used to assess discrimination. Because an independent tumor–normal test cohort was not available for final model evaluation, the reported performance should be interpreted as internal exploratory performance and may be subject to optimistic estimation. Accordingly, no claim of externally validated diagnostic performance is made [33,34].

### 2.6. Immune Infiltration and Pathway Activity Analysis

The discovery dataset (GSE13507) was used for comprehensive immune infiltration and deconvolution analyses to assess the immune microenvironment involved in the IL-6/JAK/STAT signaling axis. Four deconvolution algorithms (CIBERSORT (https://cibersortx.stanford.edu, Accessed on 14 July 2026), TIMER (https://cistrome.shinyapps.io/timer, Accessed on 14 July 2026), ssGSEA (https://www.gsea-msigdb.org/gsea/msigdb, Accessed on 14 July 2026), and MCP-counter (https://github.com/ebecht/MCPcounter, Accessed on 14 July 2026)) were used to calculate the relative abundance of important immune and stromal cell types, such as CD8+ T cells, macrophages, B cells, and dendritic cells, from bulk expression profiles. Core hub genes (IL6, IL6ST, JAK1, LIFR, OSM, CNTF, IL11, IL27RA) were then used to construct Spearman correlation matrices to investigate microenvironmental crosstalk in relation to specific immune infiltration patterns and reveal functional associations, which were displayed as hierarchical heatmaps. Due to the different immune cell deconvolution algorithms used in this study, interpretation was done based on the pattern consistency among various methods because the outputs from these algorithms can only be regarded as estimations and not as absolute quantifications of immune cell populations. A pattern that exhibited a similar trend in many methods was considered consistent, while patterns specific to particular algorithms were cautiously interpreted. The single-cell analysis was conducted as part of the exploratory secondary analysis of processed and pre-annotated expression data obtained from TISCH2 TISCH2 (https://ngdc.cncb.ac.cn/databasecommons/database/id/7538, Accessed on 15 July 2026), CancerSEA (http://biocc.hrbmu.edu.cn/CancerSEA/home.jsp, Accessed on 15 July 2026), and Human Cell Atlas databases (https://www.humancellatlas.org, Accessed on 15 July 2026). Annotations related to the particular type of cells provided by the corresponding database were utilized to analyze the distribution pattern of the chosen hub genes within major tumor and immune cells. De novo processing and analysis of raw scRNA-seq data, including preprocessing, quality control filtering, clustering, and cell-type annotation, were not conducted within the framework of this study [35,36,37].

### 2.7. Single-Cell RNA-Seq Validation

To address the challenge of cellular heterogeneity and identify the microenvironmental localization of the identified IL-6/JAK/STAT hub genes (IL6, IL6ST, JAK1, LIFR, OSM, CNTF, IL11, IL27RA), single-cell RNA sequencing (scRNA-seq) validation was incorporated through the TISCH2 (https://ngdc.cncb.ac.cn/databasecommons/database/id/7538, Accessed on 16 July 2026), CancerSEA (http://biocc.hrbmu.edu.cn/CancerSEA/home.jsp, Accessed on 16 July 2026), and Human Cell Atlas (https://www.humancellatlas.org, Accessed on 16 July 2026) frameworks. Cell-type specific expression matrices were then pseudocoded to quantify the enrichment of gene expression in the major tumor microenvironment compartments, including malignant epithelial cells, cancer-associated fibroblasts, tumor-associated macrophages, CD8+ and CD4+ T cells, endothelial cells, B cells, and natural killer cells. To substantiate the role of the hub gene panel as being mainly expressed and communicating between immune and stromal lineages, high-resolution heatmaps were created to illustrate the distribution of these genes at the cellular level [38,39,40].

### 2.8. Survival and Prognostic Analysis

The identified IL-6/JAK/STAT signaling hub genes (IL6, IL6ST, JAK1, LIFR, OSM, CNTF, IL11, IL27RA) were further evaluated in terms of clinical prognostic value and their significance in patient survival by survival analysis using clinical and expression data from the independent validation cohort (GSE13507). Patients were divided into high-risk and low-risk groups according to the normalized expression risk score of hub genes. To estimate hazard ratios (HRs) with 95% confidence intervals, Cox proportional hazards regression models were created, and Kaplan–Meier (KM) survival curves were created to compare differences in overall survival between risk strata. It is important to note that the Cox regressions performed in the current study were exploratory and failed to adequately control for clinical confounding factors, including age, tumor stage, grade, molecular subtype, or treatment status. For this reason, these Cox regressions have been performed to test univariable survival relationships [41].

## 3. Results

### 3.1. Dataset Selection and Quality Check

**Figure 1:**
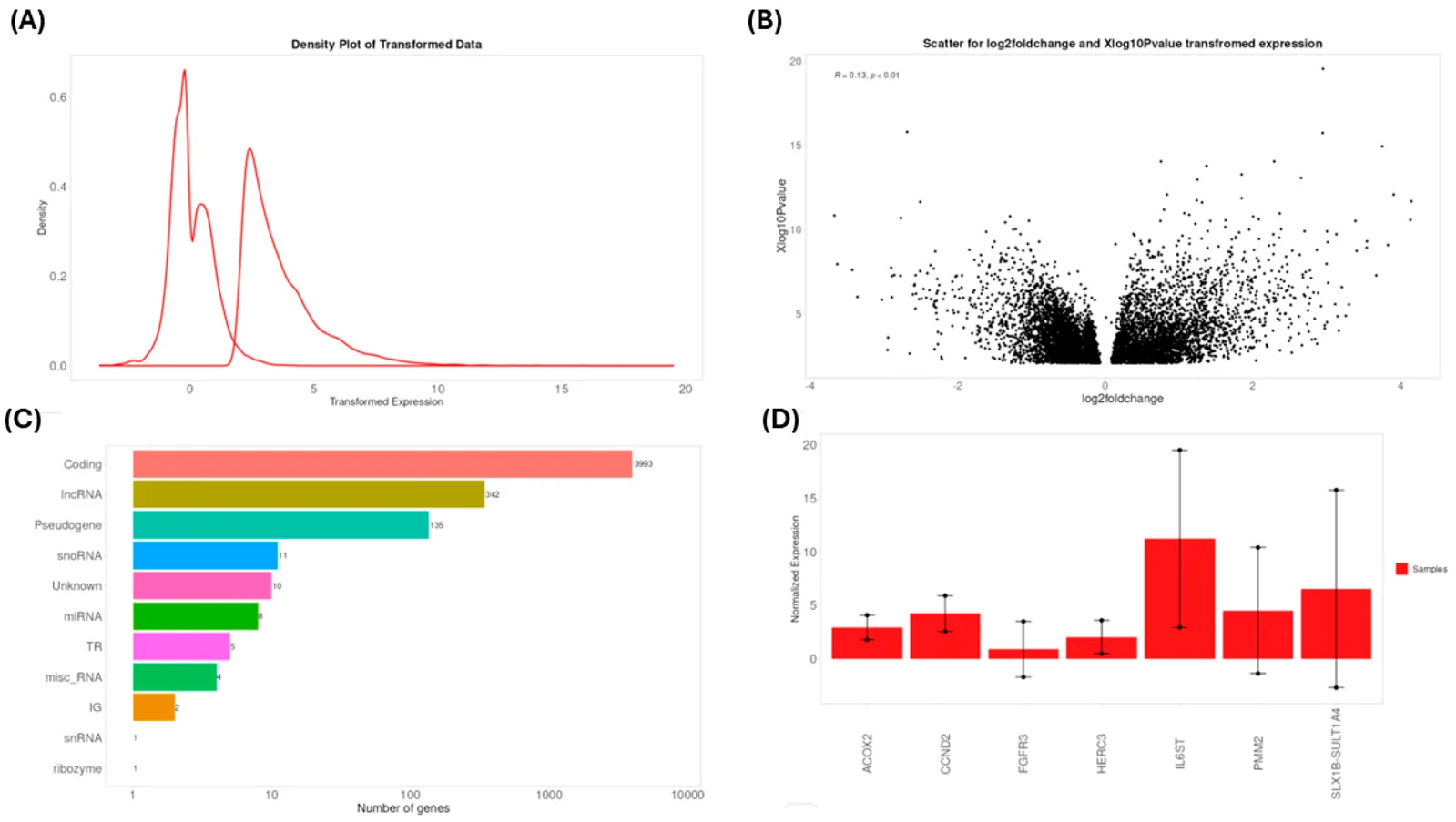
Biotype classification and gene expression characteristics. (**A**) Density plot showing the distribution of transformed gene expression values. (**B**) Scatter plot illustrating the relationship between log_2_ fold change and −log_10_-transformed *p*-values (R = 0.13, *p* < 0.01). Each point represents an analyzed expression feature. (**C**) Bar chart showing the distribution of annotated gene biotypes, including protein-coding genes, long non-coding RNAs, pseudogenes, and other RNA categories. Different colors distinguish individual biotype categories. (**D**) Normalized expression levels of selected genes, represented by red bars with error bars.

The GSE13507 dataset was employed in this downstream analysis since it showed clear distinction between urothelial carcinoma and normal bladder tissues. Upon setting up the two groups in GEO2R, it was clear that all samples showed similar expression patterns in terms of expression distribution. This indicated that the dataset was of good quality. The differential expression analysis showed that there was a unique set of genes that were significantly dysregulated in urothelial carcinoma compared to normal bladder tissue. The fact that both up- and down-regulated genes showed consistent expression in their samples indicated that the expression of these genes was disease-related. The reliability of this dataset and clear distinction between samples made it appropriate for further downstream analysis such as functional enrichment, construction of protein interaction networks, and hub gene prediction. The expression data were retrieved from the NCBI GEO database. Figure 1 below represents the data quality characteristics in the selected dataset.

### 3.2. Differential Expression Analysis

**Figure 2:**
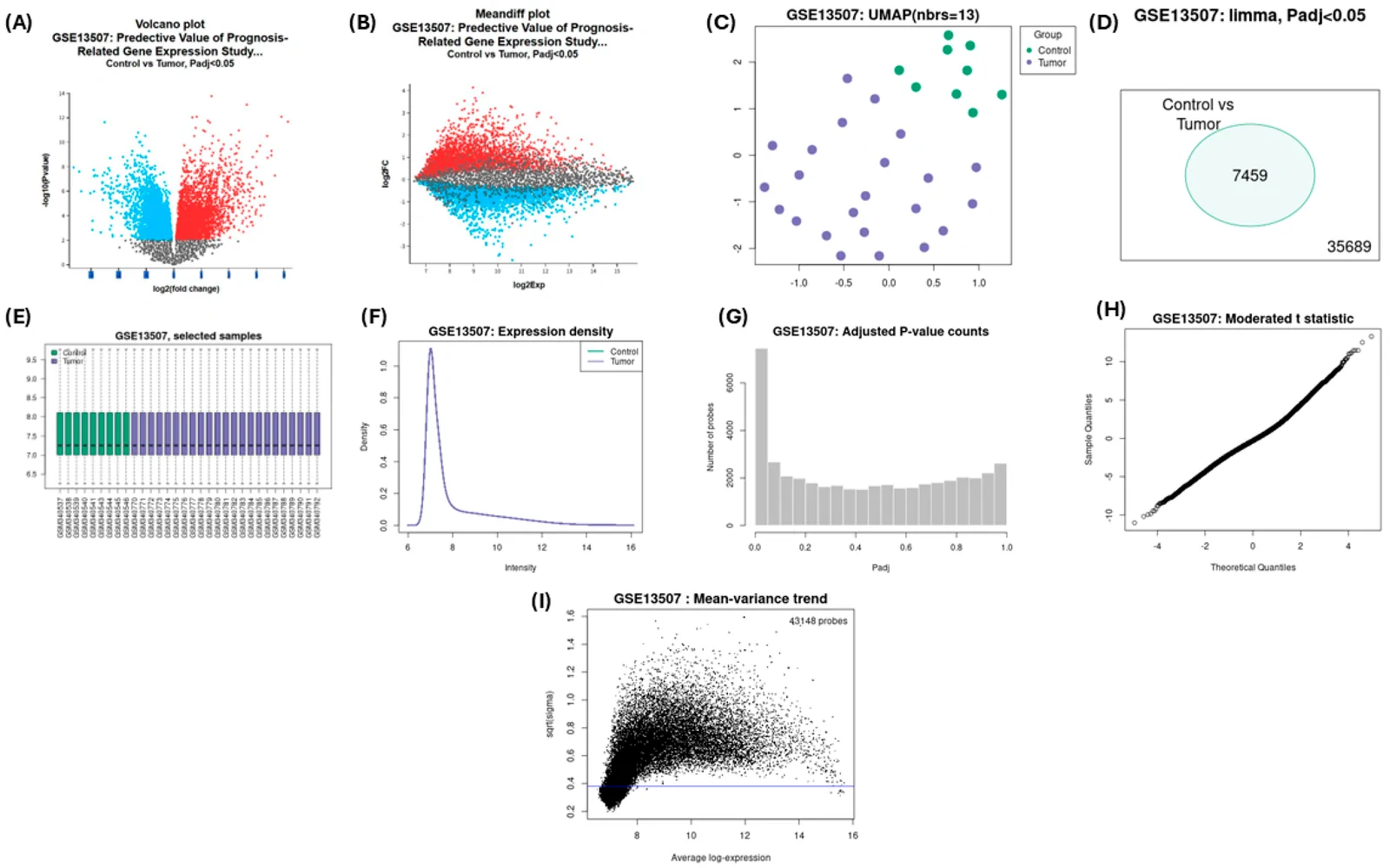
Quality analysis and identification of DEGs in GSE13507 dataset. (**A**,**B**) Volcano and mean-difference plots indicating up-regulated (red) and down-regulated (blue) genes. (**C**) UMAP clustering; (**D**) Venn diagram; (**E**,**F**) box plot and density plots; (**G**–**I**) statistical distribution plots.

By the analysis of GEO2R, urothelial carcinoma samples and normal bladder tissue from the GSE13507 dataset were analyzed. The analysis revealed a complete set of differentially expressed genes (DEGs). In total, 7459 probes demonstrated significant differential expression between control and tumor samples, as indicated by the overlap in the Venn diagram (Figure 2D). The reliability of the DEG analysis is also indicated by the volcano plot (Figure 2A) and the mean-difference plot (Figure 2B), which indicate the presence of two separate groups of DEGs based on expression levels. The adjusted *p*-values (Figure 2G) and the moderated t-statistics (Figure 2H) indicate that the differential expression is statistically significant and not due to noise. The DEG list obtained was then employed for functional analysis and the generation of a protein–protein interaction (PPI) network, with the objective of uncovering the candidate genes associated with urothelial carcinoma.

### 3.3. Functional Annotation and Pathway Analysis

The BP terms indicate that the differentially expressed genes are mainly involved in the immune response and maintaining the integrity of epithelial tissues. For example, the positive regulation of the adaptive immune response (GO:0002821) and the differentiation of T helper 17 cells (GO:0072539) indicate that the urothelial carcinoma microenvironment is dominated by the immune response. Other terms indicate the differentiation of intestinal epithelial cells (GO:0060575) and the establishment of epithelial cell apical/basal polarity (GO:0045198), which indicate changes in cellular structure that are typical in carcinomas.

Under the Molecular Function category, the terms indicate that the DE genes are mainly related to cytokine activity, particularly the binding of the interleukin-6 receptor (GO:0005138) and the activity of the interleukin-17 receptor (GO:0030368). The presence of protein tyrosine kinase activator activity (GO:0030296) and cytokine receptor activity (GO:0004896) suggests changes in intracellular signaling pathways that could promote the growth of the urothelial carcinoma. Under the Cellular Component category, the terms indicate that the DE genes are mainly related to the nuclear outer membrane (GO:0005640) and membrane rafts (GO:0045121). These findings are in agreement with the COMPARTMENTS database, where the plasma membrane signaling receptor complex and the endomembrane system have been identified as the major activity sites of the enriched proteins.

Further insights from the Jensen DISEASES and Human Phenotype Ontology databases suggest that urothelial carcinomas could have connections with rheumatoid arthritis and bone inflammatory diseases, among other conditions. The TISSUES database provides tissue-specific enrichment data showing the presence of the urothelial carcinoma proteins in the synovium and parenchyma, with the experimental data showing the presence of the proteins in the alimentary canal and the pancreatic islets.

### 3.4. PPI Network Construction and Hub Gene Identification

**Figure 3:**
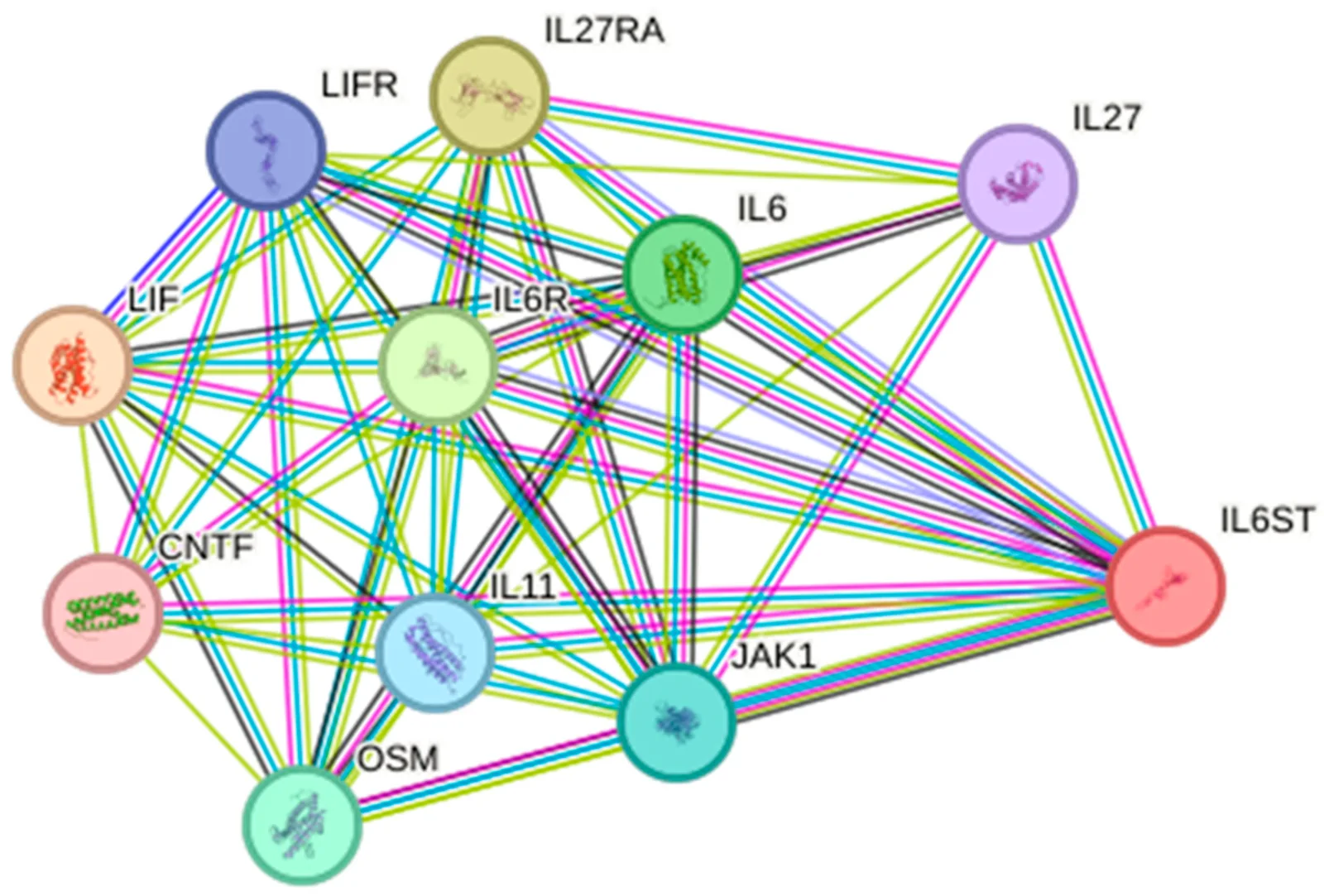
Protein–protein interaction (PPI) network of core hub genes. The network, constructed via the STRING database, illustrates the functional and physical associations between the top dysregulated proteins in urothelial carcinoma.

To identify the function of the dysregulated genes that have abnormal activity, a protein–protein interaction network was constructed using the STRING database. The network is densely connected, and there are significantly more connections than expected by chance (PPI enrichment *p*-value < 1.0 × 10^−16^). One of the most important functionally grouped regions of this complex network is centered on the IL-6 signaling pathway. The central role in signaling is played by IL6ST (gp130). The strong physical and functional interactions between IL6ST and the IL6 receptor (IL6R) and the JAK1 protein highlight the significance of the JAK/STAT pathway in the urothelial carcinoma microenvironment. The network also contains other key regulators such as LIFR, IL27RA, and ligands such as OSM and CNTF. The existence of this signaling complex indicates that these proteins could be biologically relevant players in IL-6/JAK/STAT signaling in urothelial carcinoma, but their roles need to be experimentally validated. The Figure 3 below represents the Protein–protein interaction (PPI) network of core hub genes.

### 3.5. Hub Gene and Module Detection

Using the Maximal Clique Centrality (MCC) algorithm, a primary module of the protein interaction network was generated, which is dominated by the IL-6 family of cytokines and their associated transducers. These include the genes IL6, IL6R, IL11, IL6ST, and JAK1, which have the highest scores in the top tier of the centrality scores. Table 1 below represents the results of MCODE.

**Figure 4:**
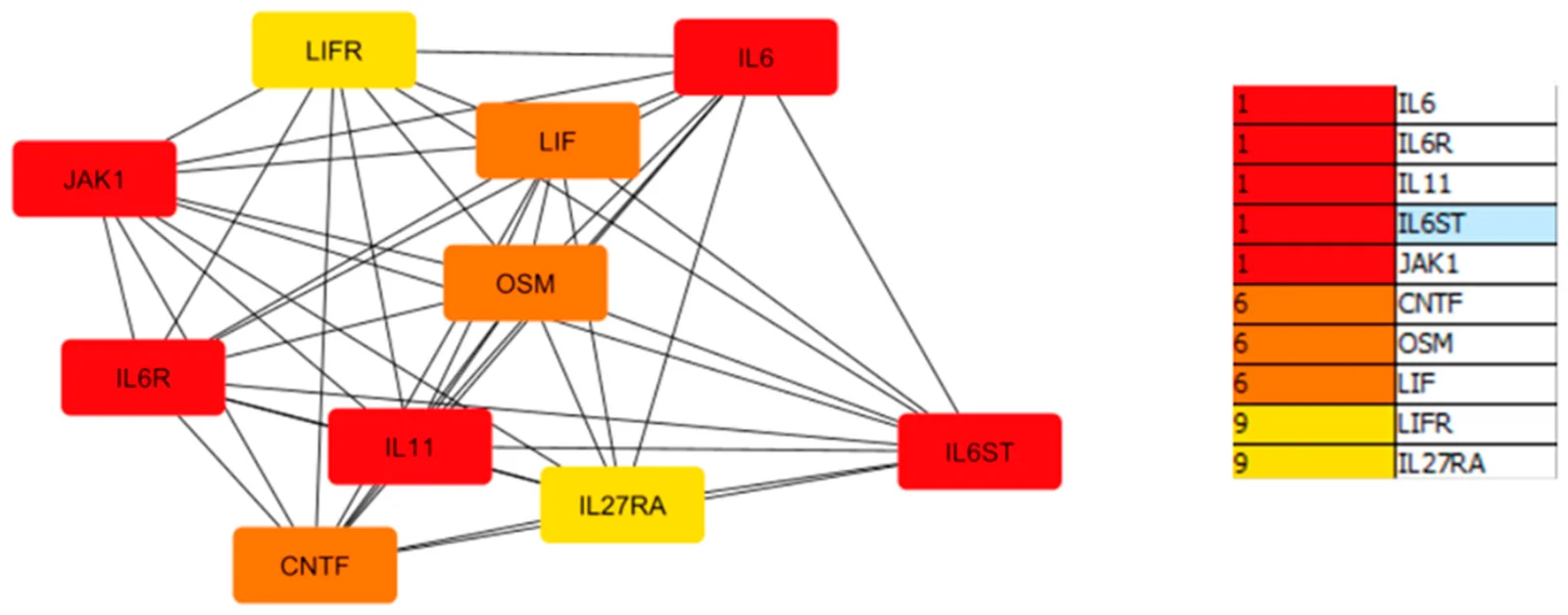
Identification of the top 10 hub genes through topological ranking. The core interaction module was extracted and analyzed using the CytoHubba plugin in Cytoscape to identify key regulatory nodes.

These are followed by the secondary genes CNTF, OSM, and LIF, which further increase the connectivity of the network. The consistency of the findings between the topological ranking and the cluster detection approaches has reinforced the notion that the IL6/JAK/STAT signaling hub is not only statistically significant in the protein interaction network, but it is consistently associated with molecular networks implicated in urothelial carcinoma. Figure 4 below represents the generated HUB Genes network by the CytoHubba Plugin used in Cytoscape.

### 3.6. Independent External Validation of Hub Genes

The independent GSE32894 cohort consisting of 308 samples of urothelial carcinoma was used to examine the expression of seven of the hub genes from the IL-6/JAK/STAT pathway. The expression of all genes was identified throughout the patient population, but levels of expression had a great deal of variation among the patients. IL6 displayed the lowest median expression compared to the cohort-centered scale (median = −0.567), while JAK1 had the highest median expression (median = 0.083). IL6 also exhibited the highest overall gene variability, with a standard deviation of 1.098 and an interquartile range of 0.999. OSM also exhibited significant interpatient variability, with some samples having very high expression. Table 2 below represents the expression characteristics of IL-6/JAK/STAT-related hub genes in GSE32894.

**Figure 5:**
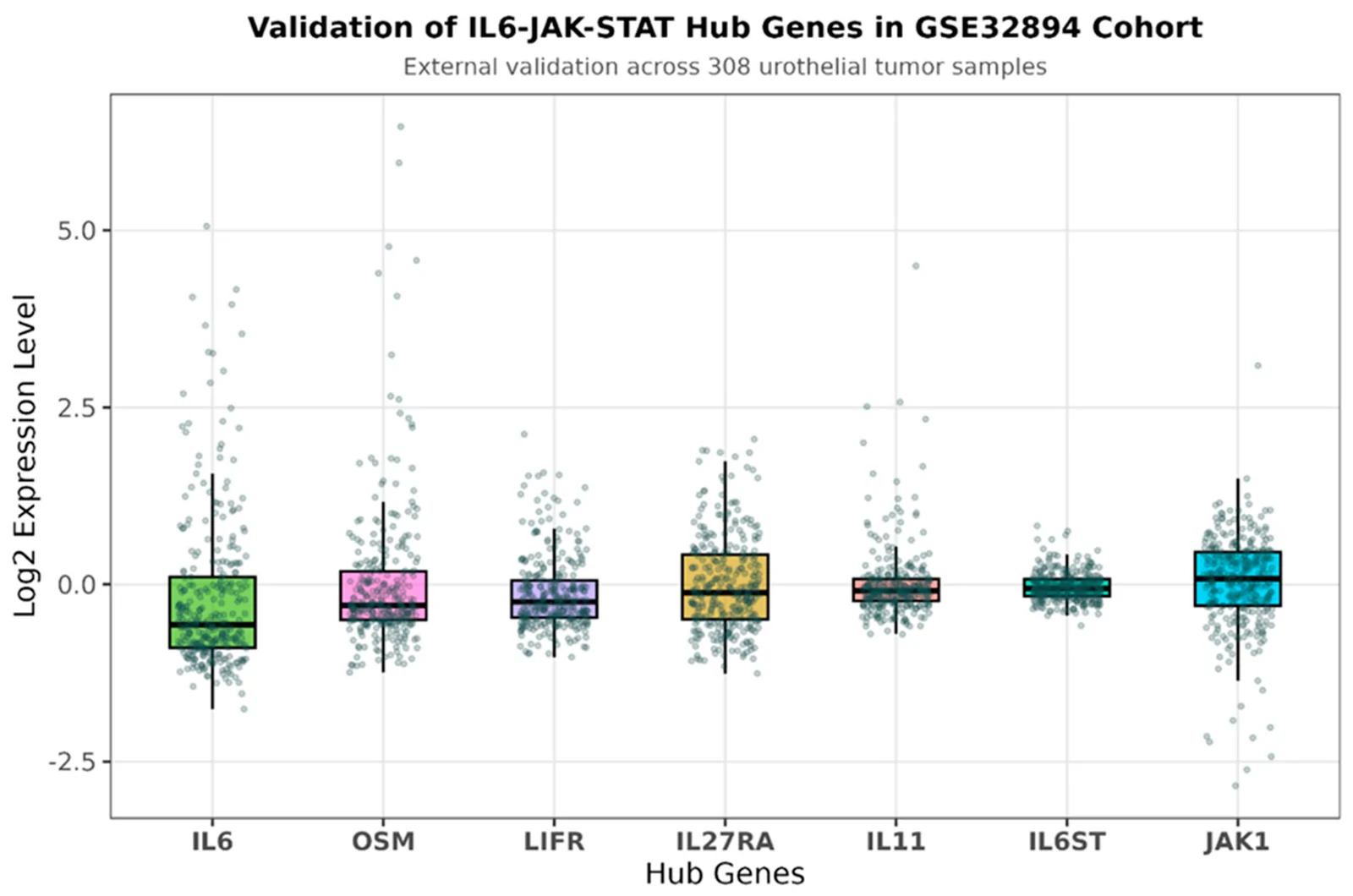
Expression distribution of IL-6/JAK/STAT-related hub genes in the independent GSE32894 urothelial carcinoma cohort. Boxplots show the cohort-centered log2 expression levels of IL6, OSM, LIFR, IL27RA, IL11, IL6ST, and JAK1 across 308 urothelial tumor samples. The central horizontal line represents the median, the boxes indicate the interquartile range, and the whiskers represent the distribution beyond the quartiles. Individual points represent patient-level expression values.

IL6ST, on the other hand, showed the most stable expression pattern, with the lowest interquartile range (IQR = 0.241) and smallest standard deviation (SD = 0.208). The distributions of the expression of IL27RA and JAK1 were also wide-ranging, and LIFR and IL11 had comparatively low levels of expression variation. Figure 5 below represents the cohort-centered log2 expression levels of IL6, OSM, LIFR, IL27RA, IL11, IL6ST, and JAK1 across 308 urothelial tumor samples.

These results validate the detection of the IL-6/JAK/STAT-related genes in an independent cohort of urothelial cancer patients and show significant patient-level heterogeneity, especially for the genes IL6, OSM, IL27RA, and JAK1. But due to the lack of a non-malignant control group in GSE32894, these results confirm expression and intertumoral heterogeneity, not expression up-regulation in the tumor itself. Therefore, direct tumor-normal validation should be done in TCGA-BLCA or another tumor-normal validation set.

### 3.7. Diagnostic Performance of Hub Genes and the Multigene Model

The diagnostic capacity of the evaluated hub genes was significantly varied by receiver operating characteristic (ROC) analysis. IL6ST demonstrated the highest discriminatory ability, with an AUC of 0.927 (95% CI: 0.891–0.963), followed closely by LIFR, with an AUC of 0.921 (95% CI: 0.853–0.989). JAK1 also showed good diagnostic performance, with an AUC of 0.843 (95% CI: 0.781–0.905). The discrimination of CNTF was moderate, with an AUC of 0.728, but the confidence interval was relatively large. Limited diagnostic performance was observed for IL27RA and IL11 (AUC = 0.646 and 0.641, respectively), while weak discrimination was observed for OSM (AUC = 0.554). IL6 did not show any useful diagnostic performance alone, with an AUC < 0.50. Table 3 below represents the diagnostic performance of individual hub genes.

**Figure 6:**
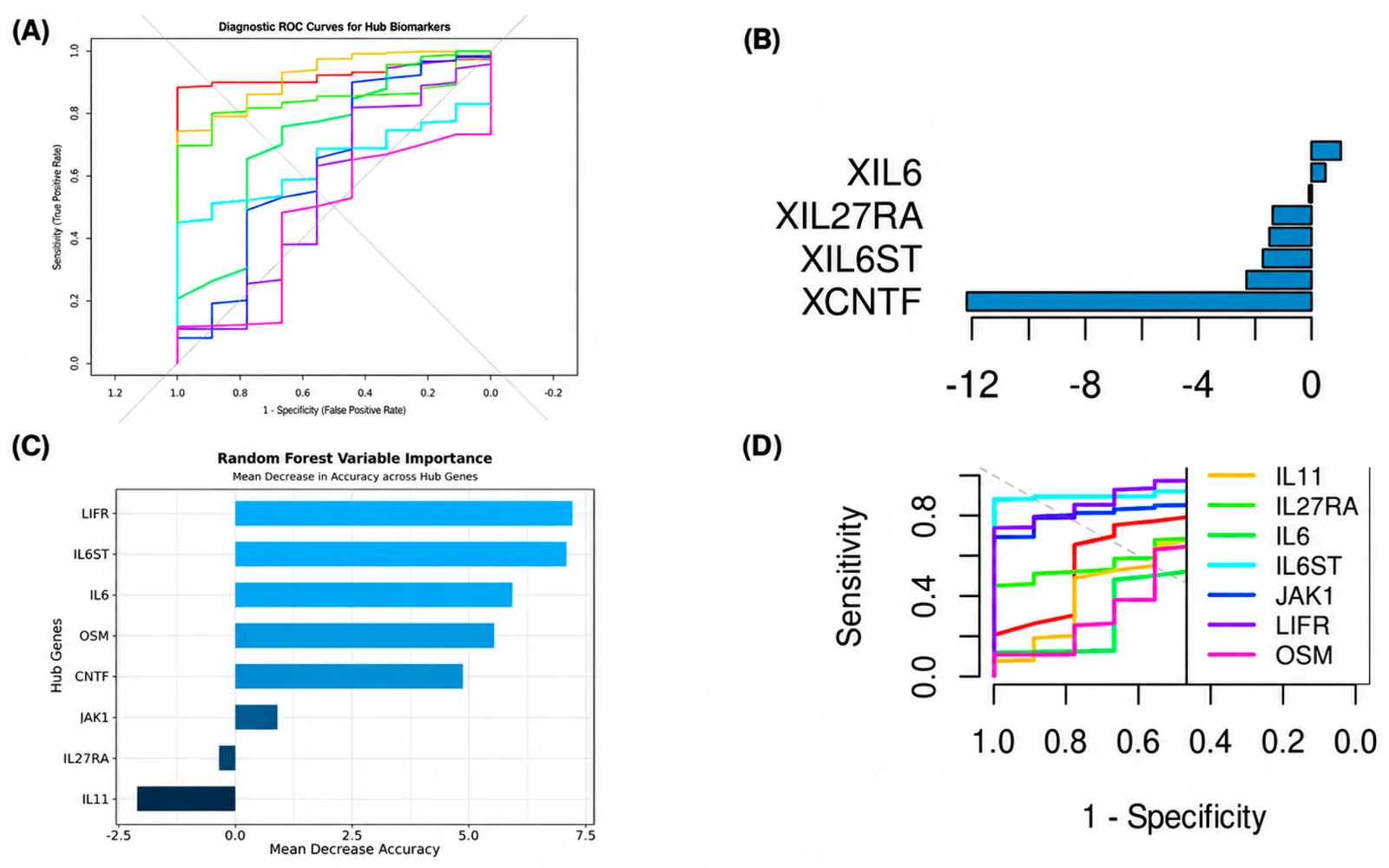
Diagnostic assessment of hub genes in urothelial carcinoma. (**A**) ROC curves of individual hub genes. (**B**) Multivariable logistic regression coefficients. (**C**) Random Forest variable importance. (**D**) Comparative ROC curves of the selected hub biomarkers.

Multivariate logistic regression showed that the candidate genes had different contributions to the total diagnostic model, with the gene CNTF having the highest negative coefficient, followed by smaller negative coefficients for the genes IL6ST and IL27RA. Positive model coefficients were observed for LIFR and IL6. Random Forest analysis also identified LIFR and IL6ST as the most informative variables; OSM and CNTF also provided relatively weak contributions to accuracy of classification; JAK1 provided a relatively weak contribution. IL27RA and IL11 were found to have negative permutation importance values, suggesting that they may not be very useful to include with the rest of the predictors. Together, these results show that IL6ST, LIFR, and JAK1 are the strongest individual markers, and the machine learning analysis points to a crucial role of IL6ST and LIFR in a multigene classifier. Figure 6 below represents the diagnostic evaluation of IL-6/JAK/STAT-related hub genes and multigene models in urothelial carcinoma. Although several molecular features demonstrated favorable discriminatory performance, these analyses did not evaluate whether the molecular signature provides incremental predictive value beyond routinely available clinicopathological variables. Therefore, the reported AUCs should be interpreted as measures of discriminatory ability within the analyzed dataset rather than evidence of established clinical utility.

### 3.8. Immune Infiltration and Hub Gene–Immune Associations

The hub gene expression was shown to have unique associations with immune cell infiltration using Spearman correlation analysis. LIFR had the highest correlation with dendritic cells (ρ = 0.60) and was correlated with NK cells (ρ = 0.52) and CD8 T cells (ρ = 0.47). IL6ST demonstrated broad positive correlations with dendritic cells (ρ = 0.55), NK cells (ρ = 0.44), CD8 T cells (ρ = 0.42), neutrophils (ρ = 0.41), and macrophages (ρ = 0.40). On the other hand, dendritic cells (ρ = 0.53) and CD8 T cells (ρ = 0.41) were also linked to JAK1. IL6 was most closely correlated with neutrophils (ρ = 0.50) and macrophages (ρ = 0.43); OSM was most closely correlated with macrophages (ρ = 0.52) and neutrophils (ρ = 0.49). In summary, there appeared to be relationships between IL-6/JAK/STAT pathway hub gene expressions and predicted immune cell infiltration, mainly with regard to dendritic cells, macrophages, neutrophils, CD8+ T cells, and NK cells. In order to avoid differences in the predictions made by the deconvolution method used, these findings can be seen as a form of computational immune association only. The mutual organization of these proteins implies their biological relevance in the signaling pathway involving IL-6, JAK, and STAT in urothelial carcinoma. Nonetheless, network centrality and computational association are not sufficient to prove functional dependence or targetability, and this needs experimental validation. Table 4 below represents the principal correlations between hub genes and immune cell infiltration.

**Figure 7:**
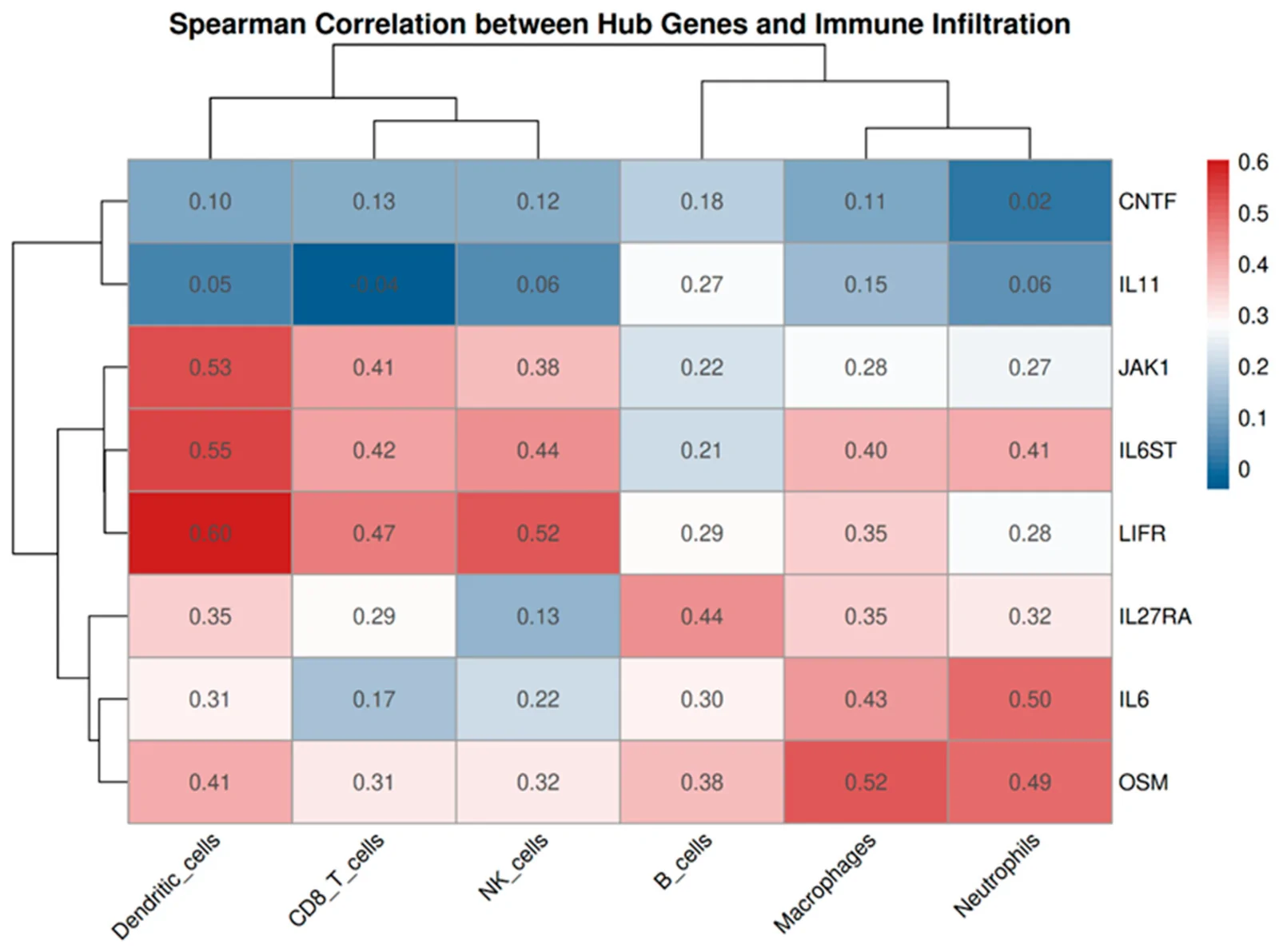
Correlation between hub genes and immune cell infiltration in urothelial carcinoma. The clustered heatmap shows Spearman correlation coefficients between hub gene expression and estimated immune cell abundance. Red indicates stronger positive correlations, whereas blue indicates weak or negative correlations.

Moderate correlations were observed with B cells, macrophages, dendritic cells, and neutrophils for IL27RA. CNTF and IL11 had a generally weak immune association, in contrast. Overall, the findings in this study suggest that there is a correlation between the IL-6/JAK/STAT-related hub genes and the immune microenvironment of UC, specifically with dendritic cells, macrophages, neutrophils, CD8 T cells, and NK cells. Correlation between hub genes and immune cell infiltration in urothelial carcinoma is given below in Figure 7.

### 3.9. Single-Cell Validation and Cellular Distribution of Hub Genes

**Figure 8:**
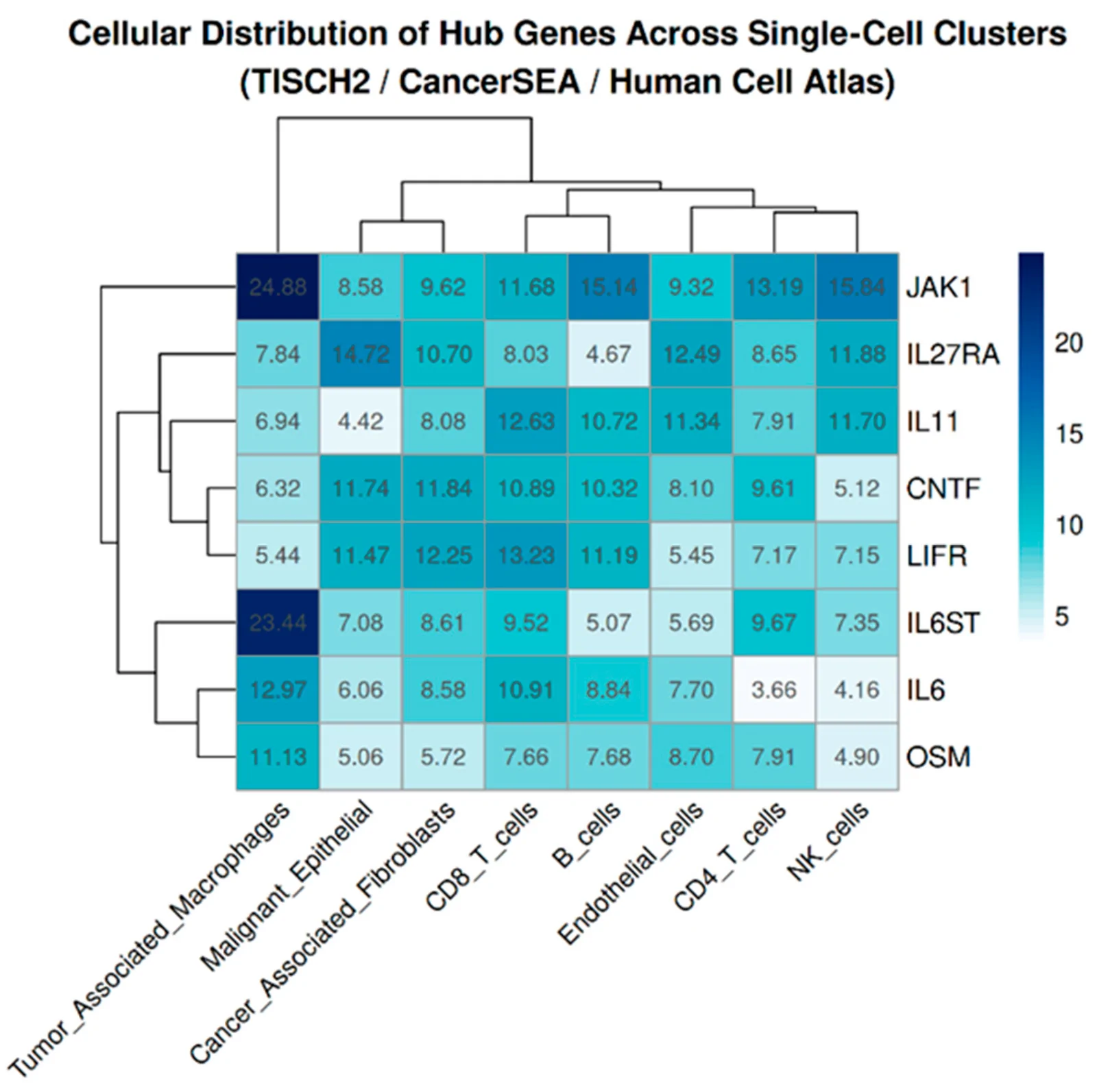
Single-cell distribution of hub genes across urothelial carcinoma cell populations. The clustered heatmap shows relative expression of IL-6/JAK/STAT-related hub genes across malignant epithelial, stromal, and immune cell populations. Higher values indicate greater cell-type-specific expression.

Single-cell analysis revealed that the expression patterns of the IL-6/JAK/STAT related hub genes were different among cell types. The expression scores for JAK1 and IL6ST were 24.88 and 23.44, respectively, and were the highest in tumor-associated macrophages. IL6 and OSM were also found to be highly enriched in tumor-associated macrophages, indicating a significant myeloid component to signaling through IL6. IL27RA was most highly expressed in malignant epithelial cells, and cancer-associated fibroblasts were most abundant in cancer-associated expression of CNTF. CD8 T cells expressed the highest levels of IL11 and LIFR. JAK1 was also up-regulated in B cells and NK cells, suggesting widespread expression in immune cells. Figure 8 below represents the single-cell distribution of hub genes across urothelial carcinoma cell populations.

### 3.10. Survival and Prognostic Significance of Hub Genes

However, for the high- and low-risk hub gene groups, there was significant overlap in the survival curves (Figure 1), and no distinct separation could be observed during the follow-up. In the same manner, none of the eight hub genes was significantly correlated with OS in univariable Cox regression. JAK1 showed the strongest trend toward increased mortality risk (HR = 1.16, 95% CI: 0.95–1.42, *p* = 0.149), followed by IL6ST (HR = 1.19, 95% CI: 0.74–1.90, *p* = 0.472). All confidence intervals, however, were intercepting 1.0, and all *p*-values were greater than 0.05. The findings fail to suggest any significance of the hub genes identified in terms of their predictive power regarding the overall survival in the investigated cohort. As the analysis of survival was not conducted in full by taking into account various clinical covariates, the prognostic role cannot be inferred from the current findings. Table 5 below represents the Cox regression analysis of hub genes for overall survival.

**Figure 9:**
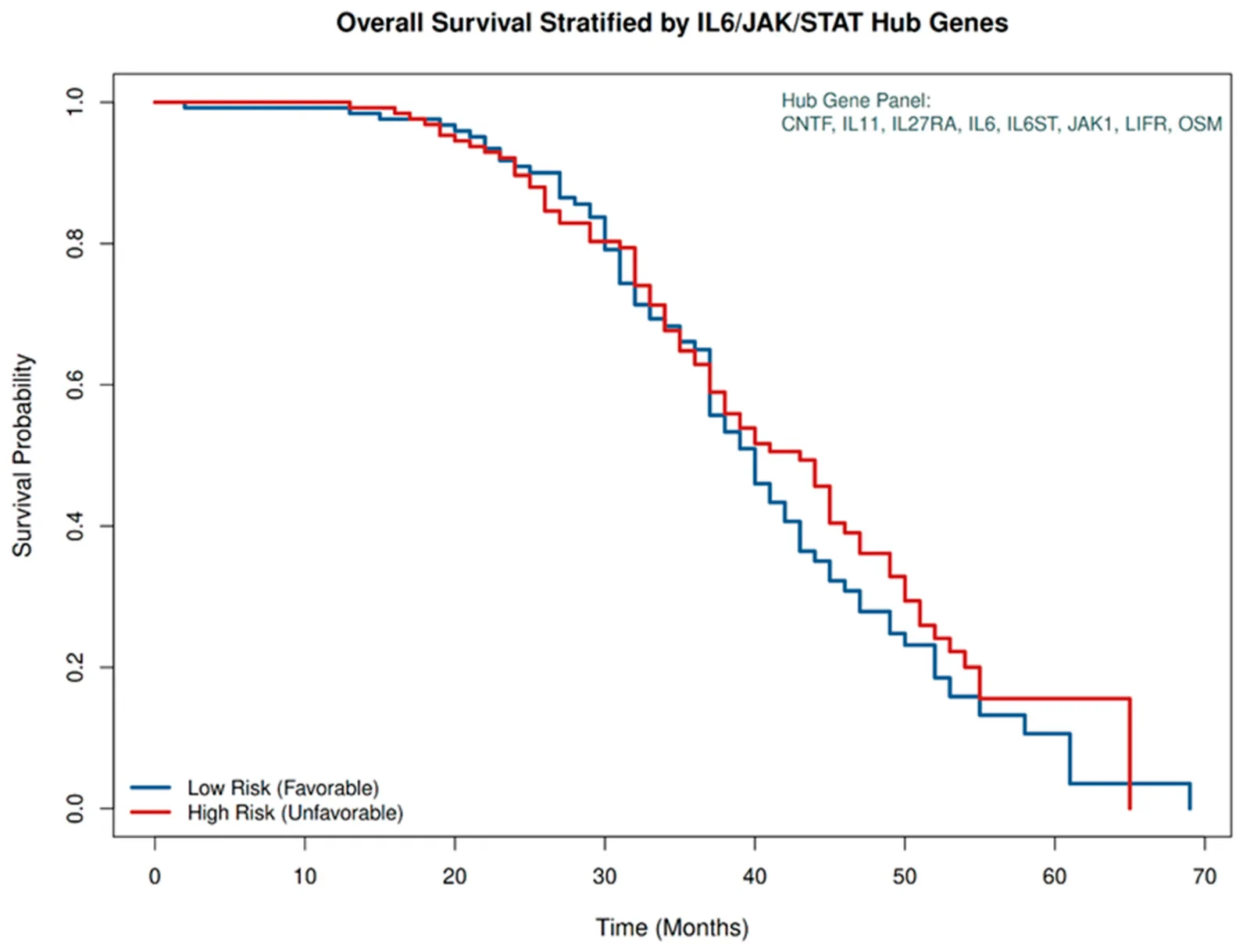
Overall survival according to the IL-6/JAK/STAT hub gene signature. Kaplan–Meier curves compare patients classified into high- and low-risk groups. The substantial overlap between the curves indicates limited prognostic discrimination.

These results suggest that the genes identified might be biologically and diagnostically significant but are not predictive of overall survival in the subset of patients examined in this study. Figure 9 below represents the overall survival according to the IL-6/JAK/STAT hub gene signature.

## 4. Discussion

This study aimed to determine if transcriptomic and network-based analysis could reveal biologically relevant hub genes, potential diagnostic candidates, and immune-associated regulatory mechanisms in urothelial carcinoma. The network of IL-6/JAK/STAT genes, including IL6, IL6ST, JAK1, LIFR, OSM, CNTF, IL11, and IL27RA, was repeatedly identified in all types of analyses performed here: differential-expression, enrichment, PPI, external cohort, machine learning, immune-infiltration, and single-cell. The hub gene panel had little prognostic significance for overall survival, whereas IL6ST, LIFR, and JAK1 had the best diagnostic performance [42,43,44,45].

The findings indicate that the genes identified are more strongly linked to the biology of the tumor and to the regulation of the immune system and diagnostic discrimination than to survival time. LIFR, IL6ST, JAK1, IL6 and OSM have strong correlations with dendritic cells and macrophages, neutrophils, CD8 T cells and NK cells, suggesting that the pathway may reflect tumor cells interacting with the immune microenvironment. Single-cell analysis also revealed that these genes were present in the malignant, stromal, and immune compartments and were especially up-regulated in tumor-associated macrophages (TAMs), particularly in the case of JAK1, IL6ST, IL6 and OSM [46,47].

Signaling processes other than the transcriptional abundance of a protein may also link signaling pathways to a particular phenotype of the cell. Zhong et al. found that the activation of the Wnt/β-catenin signaling pathway increases the activity of TCF4/YTHDF1. The function of YTHDF1 is to translate GPX4 and SLC7A11, which prevent ferroptosis. This example is evidence that regulation of signaling processes influences the oxidative-stress and cell death programs through post-transcriptional regulation. In the case of urothelial carcinoma, a similar regulatory relationship may exist between cytokine/JAK/STAT signaling and cellular stress programs and tumor-microenvironment communication. However, this interaction was not studied in the present study and should be regarded as a hypothesis [48].

The results are overall similar to those from previous reports on the roles of immune and cytokine signaling in bladder cancer biology. Previous studies have also correlated the immune cell infiltration, tumor heterogeneity, and disrupted signaling pathways with the progression of urothelial carcinoma. To build on these observations, the present study combines the concepts of network topology, diagnostic modeling, immune deconvolution, and single-cell localization. None of the individual hub genes had significant associations with survival in the current survival analysis; however, this is not the case for studies that have found strong prognostic signatures of multiple genes working together. The relationship between molecular signatures and the immune microenvironment should be interpreted cautiously. EMT-associated results must be viewed in context, because an increase in gene expression of individual EMT-associated genes may not be a clear indicator of EMT program activation. In the case of urothelial carcinoma, Khan et al. found correlations between SNAIL/SLUG and TWIST expression and clinicopathological features, including muscle invasion, but focused on expression of individual EMT markers. More generally, Ramalingam et al. stated that EMT is a dynamic and heterogeneous phenomenon consisting of coordination of transcriptional factors and signaling pathways, such as SNAIL, SLUG, TWIST, ZEB, TGF-β/SMAD, and Wnt/β-catenin signaling. Hence, any EMT-associated features identified in the current study should be considered only as marker-specific or pathway-related data, but not as confirmation of the entire EMT program [49,50]. Duan et al. reported that a TGF-β signaling-related lncRNA risk signature in glioma was associated with distinct immune-infiltration patterns and predicted immunotherapy response, illustrating how transcriptomic signatures can capture differences in the tumor immune landscape. However, such computational associations do not by themselves establish direct mechanistic regulation of immune cells. Similarly, the associations observed here between IL-6/JAK/STAT-related hub genes and estimated immune cell infiltration should be considered predictive and biological associations rather than evidence of causality. Functional and spatial studies will be required to determine whether these genes directly influence immune cell recruitment and activation, or tumor–immune interactions [51,52,53,54,55].

The discovery analysis was predominantly based on retrospective public data, and GSE32894 did not contain non-malignant controls, so it confirmed that expression was heterogeneous, but not that it was increased in tumors. Diagnostic models need to be better externally validated, calibrated, and tested in the future. Single-cell results were derived from public resources, and immune infiltration was estimated using a computational approach. Confirming protein-level and functional experiments were also not conducted. Moreover, the biological significance of the identified hub genes is based on computational convergence of evidence and not causality proof. Differentiation in expression, network centrality, diagnostic classification, immune cell association, and cell type specificity in expression serve as supportive evidence that the hub genes are associated with a disease state but are not evidence for direct participation in urothelial carcinoma. Experimental differences in the platforms, samples, and clinical annotations used in each publicly available cohort could contribute to both technical and biological heterogeneity. The lack of batch correction across the cohorts is due to the independent analysis of each dataset.

The most promising candidates (IL6ST, LIFR, JAK1, among others) should be validated in independent TCGA-BLCA and other tumor-normal sets in future studies. These genes will require prospective tissue, urine, or blood validation before they can be used as clinically useful biomarkers. Their functional roles should also be tested in experimental studies by knocking down genes and inhibiting pathways and in immune cell co-culture models. The combination of proteomics, spatial transcriptomics, and cell-cell communication analysis would further elucidate the role of the IL-6/JAK/STAT network in the progression of UC and response to treatment. It is crucial to highlight the difference between computational identification of immune signatures and evidence of their functional manipulation to remodel the microenvironment. In the case of Wang et al., the researchers used an association between an immunosuppressive granuloma microenvironment and a tested therapeutic intervention and thus demonstrated the link between the findings regarding the microenvironment and intervention for its modification. However, the patterns of immune genes found in the current research were computationally identified, analyzed with respect to their correlations with immune cells, and examined at the single-cell level. Thus, they only suggest the presence of potential biological links, not proving the ability of modification of the IL-6/JAK/STAT-related genes to change the microenvironment [56].

It must be noted that the current investigation did not model or predict anticancer drug response. As a result, the hub genes discovered here should not be regarded as the validated predictors of therapy sensitivity. Transcriptomics and machine learning models may serve as the basis for drug response prediction. In particular, Wang et al. combined transcriptomic data with pharmacological databases like GDSC and CCLE. Ramalingam et al., in their turn, used the approaches of bioinformatics and machine learning screening in order to find molecular inhibitors. These examples show the possible effectiveness of such computational approaches; however, at the same time, they demonstrate the necessity of additional experimental validation. The infiltration of immune cells was determined computationally rather than experimentally, and the estimates could vary based on the choice of deconvolution algorithm, reference genes used, and cell content of the samples. Therefore, results showing agreement between the methods were deemed more significant, whereas those that showed discrepancies were viewed critically. The associations would need to be validated using flow cytometry or other techniques for measuring the cells [48,57].

The translation of computationally predicted biomarkers into clinically relevant biomarkers needs more validation beyond performance within a discovery cohort. The importance of external validation was shown in the study by Zhang et al., where deep learning was used to integrate histopathology image features and nuclear segmentation features to predict MSI and TMB and evaluate model performance across different centers. This type of study underscores the necessity of external validation to determine whether the model’s performance is consistent across populations and datasets. Thus, the diagnostic performance seen for IL6ST, LIFR, and JAK1 in our current study must be viewed as preliminary evidence of biomarker utility, not evidence of clinical utility [58].

## 5. Conclusions

This study reveals an IL-6/JAK/STAT-related gene network that has significant biological and diagnostic relevance in UC and identifies three most promising candidates: IL6ST, LIFR, and JAK1. These genes were not independent predictors of overall survival, but there was a strong association between these genes and immune cell infiltration and their unique distribution across malignant, stromal, and immune compartments, indicating a potential to influence the tumor microenvironment. The results indicate the candidacy of IL6ST, LIFR, and JAK1 as diagnostic biomarkers and biologically interesting genes for future studies. It is to be noted that the current study does not prove the targetability of these molecules, and hence further validation studies are needed to assess their possible therapeutic role. These showed promising discriminatory performance and therefore represent candidate diagnostic biomarkers for further validation. Their incremental value beyond established clinicopathological predictors and their potential clinical utility remain to be determined in independent, clinically annotated cohorts.

## Figures and Tables

**Table 1:** The scoring of MCODE on the identified NODES from Cytoscape.

Gene Name	Score	Status
CNTF	7.8222	Clustered
OSM	7.8222	Clustered
LIF	7.8222	Clustered
IL27RA	8.0000	Clustered
IL11	7.8222	Clustered
JAK1	7.8222	Clustered
IL6	7.8222	Clustered
IL6R	7.8222	Clustered
IL6ST	7.8222	Clustered
LIFR	8.0000	Seed
IL27	5.7857	Unclustered

**Table 2:** Expression characteristics of IL-6/JAK/STAT-related hub genes in the GSE32894 validation cohort.

Hub Gene	Mean Expression	Standard Deviation	Median Expression	Interquartile Range
IL6	−0.199	1.098	−0.567	0.999
OSM	0.003	1.008	−0.296	0.685
LIFR	−0.127	0.524	−0.245	0.522
IL27RA	0.005	0.671	−0.118	0.916
IL11	0.011	0.523	−0.089	0.312
IL6ST	−0.032	0.208	−0.056	0.241
JAK1	0.028	0.671	0.083	0.758

**Table 3:** Diagnostic performance of individual hub genes.

Gene	AUC	95% Confidence Interval
IL6ST	0.927	0.891–0.963
LIFR	0.921	0.853–0.989
JAK1	0.843	0.781–0.905
CNTF	0.728	0.538–0.917
IL27RA	0.646	0.548–0.743
IL11	0.641	0.419–0.862
OSM	0.554	0.329–0.780
IL6	0.463	0.283–0.643

**Table 4:** Principal correlations between hub genes and immune cell infiltration.

Hub Gene	Strongest Immune Cell Association	Spearman ρ	Correlation
LIFR	Dendritic cells	0.60	Strong positive correlation
IL6ST	Dendritic cells	0.55	Moderate-to-strong positive correlation
JAK1	Dendritic cells	0.53	Moderate positive correlation
OSM	Macrophages	0.52	Moderate positive correlation
IL6	Neutrophils	0.50	Moderate positive correlation
IL27RA	B cells	0.44	Moderate positive correlation
IL11	B cells	0.27	Weak positive correlation
CNTF	B cells	0.18	Weak positive correlation

**Table 5:** Cox regression analysis of hub genes for overall survival.

Gene	Hazard Ratio	95% Confidence Interval	*p*-Value
CNTF	1.07	0.21–5.43	0.939
IL11	1.12	0.82–1.52	0.486
IL27RA	1.08	0.84–1.38	0.545
IL6	1.08	0.94–1.24	0.281
IL6ST	1.19	0.74–1.90	0.472
JAK1	1.16	0.95–1.42	0.149
LIFR	1.09	0.92–1.31	0.327
OSM	1.03	0.79–1.34	0.842

## Data Availability

All the data generated in this research work has been included in this manuscript.
